# *In vitro* activity of anti-rheumatic drugs on release of pro-inflammatory cytokines from oral cells in interaction with microorganisms

**DOI:** 10.3389/froh.2022.960732

**Published:** 2022-09-02

**Authors:** Alexandra Stähli, Carina Scherler, Graziano Zappalà, Anton Sculean, Sigrun Eick

**Affiliations:** Department of Periodontology, School of Dental Medicine, University of Bern, Bern, Switzerland

**Keywords:** periodontitis, rheumatoid arthritis, anti-inflammatory drugs, proinflammatory cytokines, oral cells

## Abstract

Periodontitis patients suffering concomitantly from rheumatoid arthritis (RA) often present with less inflamed periodontal tissues due to the ongoing anti-rheumatic therapy. This *in vitro* study was aimed to analyze whether anti-inflammatory drugs used in the therapy of RA can modulate the release of IL-8 and IL-1β by professional and non-professional immune cells stimulated with microorganisms. Periodontal ligament (PDL) fibroblasts, monocytic MONO-MAC-6-cells, and gingival keratinocytes were exposed to ibuprofen, prednisolone, and methotrexate with and without lysates of *Fusobacterium nucleatum* or *Candida albicans*. Supernatants were obtained and the levels of interleukin(IL)-8 and IL-1β (only MONO-MAC-6) were quantified. The addition of *F. nucleatum* lysate resulted in the strongest release of proinflammatory cytokines by PDL fibroblast and MONO-MAC-6 cells, while the modification by the tested anti-rheumatic drugs was only minor. After stimulation of the MONO-MAC-cells with *F. nucleatum*, prednisolone increased the release of IL-8, whereas methotrexate decreased the level. Anti-inflammatory drugs increased the adherence of *C. albicans* to epithelial cells. In patients with RA, the reduction of the microbial load in subgingival biofilm (biofilm removal) is of major importance; however, the intake of inflammatory drugs may interfere with the inflammatory response.

## Introduction

Periodontitis is one of the most common diseases worldwide with a prevalence of 11% for its severe form [1]. It is a chronic disease resulting from the inflammatory response to a dysbiotic microbiota in the subgingival biofilm that eventually leads to the destruction of teeth-surrounding tissues [2]. *Fusobacterium nucleatum*, a common opportunistic bacteria, acts as a bridging bacterium between symbiotes and pathogens and is generally thought to contribute to bacterial pathogenicity [3]. The inflammasome activation is crucial in the pathogenesis of periodontal disease and it includes the maturation of the pro-inflammatory cytokines interleukin (IL)-1β and IL-18 [4]. IL-8 (IL-8/CXCL9) is a member of the CXC chemokine family, it attracts neutrophils and stimulates the release of neutrophil granules [5]. The expression of IL-8 increases with the severity of periodontal disease [6]. Meanwhile, it is well known that periodontitis does not only locally affect the periodontium. There are many associations with systemic pathologies where periodontitis may influence the progression or therapy of the systemic disease and vice versa. Diabetes mellitus was among the first systemic conditions showing a clear correlation with attachment loss [7]. Another disease that stepped into the focus of research is rheumatoid arthritis (RA). A recent large analysis of health insurance data including more than 500,000 participants in South Korea showed a higher prevalence of periodontitis in individuals with RA (19.6%) than without (16.6%) and also more cases with RA (6.2%) in patients with periodontitis than in those without (5.2%) [8].

RA is a systemic inflammatory disease characterized by joint stiffness, pain, and swelling. Antibodies against rheumatoid factor and citrullinated proteins are detectable in most patients [9]. Numerous inflammatory cells infiltrate the interstitium between cartilage and bone. Concomitantly, higher concentrations of IL-1, IL-6, IL-8, IL-10, and MCP-1 are observed which may be associated with neutrophil autophagy—a phenomenon that might play a role in the pathogenesis of RA [10, 11]. In the 70s and 80s, two inflammatory mediators were discovered to be relevant for bone and tissue destruction in the course of RA: IL-1 and tumor necrosis factor (TNF). While IL-1 is locally active, TNF plays a more prominent part systemically. The treatment of RA is scheduled to control the inflammation and lower disease activity by reducing Il-1 and TNF release [12]. Basically, disease-modifying anti-rheumatic drugs (DMARDs) and nonsteroidal anti-inflammatory drugs (NSAIDs) are applied [9]. Here, we focused on three widely prescribed drugs that belong to different classes; methotrexate, one of the DMARDs, decreases inflammation and counteracts tolerance of TNF inhibitors [12, 13]; ibuprofen, a NSAID, is able to limit local inflammation through reversible inhibition of the cyclooxygenase enzymes COX-1 and COX-2 [13]; and finally prednisolone, a glucocorticoid that suppresses the migration of polymorphonuclear leukocytes and reverses increased capillary permeability [14].

*Porphyromonas gingivalis*, a major pathogen in periodontitis, has been widely discussed as a possible link between the two diseases since its peptidyl arginine deiminase can citrullinate peptides, and in arthritis patients, increased antibodies against *P. gingivalis* have been found [15, 16]. However, in a recent murine model, other bacteria have come into focus. Complex analyses of the microbiota show *F. nucleatum* to be enriched in periodontitis vs. periodontal health [17]. In a murine model, it has been demonstrated that inoculation with *F. nucleatum* or *Aggregatibacter actinomycetemcomitans* alone spurred the onset and progression of arthritis [18]. And it was mostly bacterial DNA from *F. nucleatum* that was detected in the synovial fluid of arthritis patients [19].

In our recent studies on the association of RA with periodontal disease, tooth loss was not associated with the typical signs of periodontitis such as high probing pocket depth and bleeding on probing [16, 20, 21]. This might be a result of the longtime intake of anti-rheumatic drugs. Therefore, the question arose whether this medication might interfere with the periodontal inflammatory response. Further, as *Candida albicans* was highly present in the subgingival biofilm samples of RA patients (which was not the case in periodontally healthy and periodontitis patients without underlying systemic disease), we also included *C. albicans* in this study.

The research questions to be answered in the present *in vitro* study were (i) if anti-inflammatory drugs used in the therapy of RA patients can modulate the release of IL-8 and IL-1β by professional and non-professional immune cells being of relevance in the oral cavity and (ii) how the interaction of microorganisms with these cells is being influenced by anti-inflammatory drugs.

## Materials and methods

### Cells

Three different cells were used in the assays: periodontal ligament (PDL) fibroblasts; MONO-MAC-6-cells (DSMZ no. ACC 124), a monocytic cell line of human origin; and telomerase-inactivated gingival keratinocyte (TIGK) cells (ATCC-CRL-3397). The PDL fibroblasts originated from three different donors. PDL fibroblasts were collected from extracted teeth. Before, patients had agreed for using their cells for research purposes and signed a written consent. This procedure is in accordance with the guidelines set by the Cantonal ethical committee KEK. As the obtained biomaterials were categorized as “irreversibly anonymized,” no previous approval was necessary. The method was used as described before [22].

The cell cultivation media were DMEM for PDL fibroblasts and RPMI 1640 medium for MONO-MAC-6 cells, both supplemented with 10% fetal bovine serum (FBS; all Invitrogen; Carlsbad, CA, USA). For TIGK cells, Keratinocyte Growth Medium (KGM-Gold; Lonza, Basel, Switzerland) was used.

### Anti-rheumatic drugs

The following drugs and concentrations (final) were used in the assays.

- ibuprofen (Brufen 600 mg, BGP Products GmbH, Baar, CH): 2, 10, and 50 μg/ml
- prednisolone (Spricort 20, Spirig Health Care AG, Egerkingen, CH): 20, 100, and 500 ng/ml
- methotrexate (Methotrexat Farmos 10 mg, Orion Pharma AG, Zug, CH): 1, 5, and 25 μg/ml.

The concentrations were chosen based on reported concentrations in plasma or tissue [23–25]. Cytotoxicity was tested using trypan blue exclusion tests for the applied concentrations.

When starting experiments, a dilution series of 1:4 of the drugs in phosphate-buffered saline (PBS) was made. The first tube contained 10-fold of the highest concentrations. The respective negative control was PBS.

### Microorganisms

Lysates of *F. nucleatum* ATCC 25586 and *C. albicans* ATCC 76615 were prepared. A suspension OD600 = 1 in PBS was exposed to 20 min ultrasonication with a power of 280 W; thereafter, the mixture was centrifuged at 10,000 *g* at 20°C for 10 min. The supernatant was obtained and filtrated by using a pore size of 0.4 μm. This corresponded to a multiplicity of infection (MOI) of ~20:1 (bacteria:cells). Of note is that cells were stimulated rather with bacterial components than entire bacterial cells.

In experiments with TIGK cells, microbial cells of *C. albicans* ATCC 76615 were suspended in PBS (OD 600 nm = 0.2) resulting in a MOI of ≈5:1 (fungi:cells).

### Methods MONO-MAC-6-cells

MONO-MAC-6-cells were centrifuged for 5 min at 250 *g*. The supernatant was removed and the cells were washed two times with PBS. Thereafter, they were adjusted to a density of 10^6^/ml in RPMI 1610 media with 0.5% FBS. The cell suspension was mixed with microbial lysate and one of the drug solutions in a ratio 8:1:1 of which 1 ml was then pipetted per well in a 24-well plate. Each of the drug-microbes-cell suspensions was pipetted to a well plate. The plates were incubated for 18 h before the suspension was transferred to 1.5 ml tubes and centrifuged at 10,000 *g* for 5 min at 20°C. The supernatants were obtained and stored at −80°C until assayed by ELISA.

### Methods PDL fibroblasts

Before the experiments, PDL fibroblasts were transferred to 24-wells plates and grown to confluence. The cell cultivation medium was removed. The cells were washed two times with PBS and 1 ml of one of the drug-microbes-cell cultivation media (ratio 1:1:8) was added. Each of the drug-microbes suspension was pipetted to a well plate with seeded PDL fibroblasts (As before, the FBS concentration was reduced to 0.5% in the cell cultivation media). Cells were seeded at 3 × 10^5^/cm^2^. No more than five passages were used for the experiments. The further procedure followed those of the MONO-MAC-6-cells. All experiments were conducted in triplicates and in three independent repetitions.

### Methods TIGK cells

TIGK cells were adjusted to 10^5^/ml cell cultivation media and every 1 ml was transferred to a well of a 24-well plate 24 h before starting the experiments. On the day of the experiment, cells were checked for confluency. Then, they were washed two times, before 1 ml of one of the drug-microbes-cell cultivation media (ratio 1:1:8) was added. Each of the drug-microbes suspension was pipetted to a well plate with seeded TIGK cells. After an incubation time of 6 h, media were removed and treated as described before (centrifuging, storing of the supernatants at −80°C).

Then, the cells were washed two times with PBS to remove non-adherent *C. albicans* cells. Thereafter, 1 ml ice-cold dH_2_O/well was added. The plates were left in place for 15 min, after intensive mixing, and the suspension was plated on agar plates to determine the colony forming units of adhered (incl. invasive) *C. albicans*.

### ELISA

In preliminary tests, the suitable biomarkers were selected. All cells released IL-8. In addition, from supernatants obtained after interaction with MONO-MAC-6-cells, the levels of IL-1β were assessed.

Commercially available ELISA kits (R&D Systems, Minnesota, MN, USA) were used according to the manufacturer's instructions. The detection levels were 1 pg/ml for both IL-8 and IL-1β.

### Statistical analysis

Data were compared by using software SPSS 24.0 (IBM, Armonk, NY, USA). One-way ANOVA with *post-hoc* Bonferroni was applied. Beforehand, the assumption of normally distributed data was confirmed using skewness and kurtosis statistics. In *post-hoc* analyses, only comparisons of the drug with the respective control of the microbes or at defined drug concentrations with the cells without microbes were considered. The level of statistical significance was set at *p* < 0.05.

## Results

### MONO-MAC-6 cells

**Figure 1:**
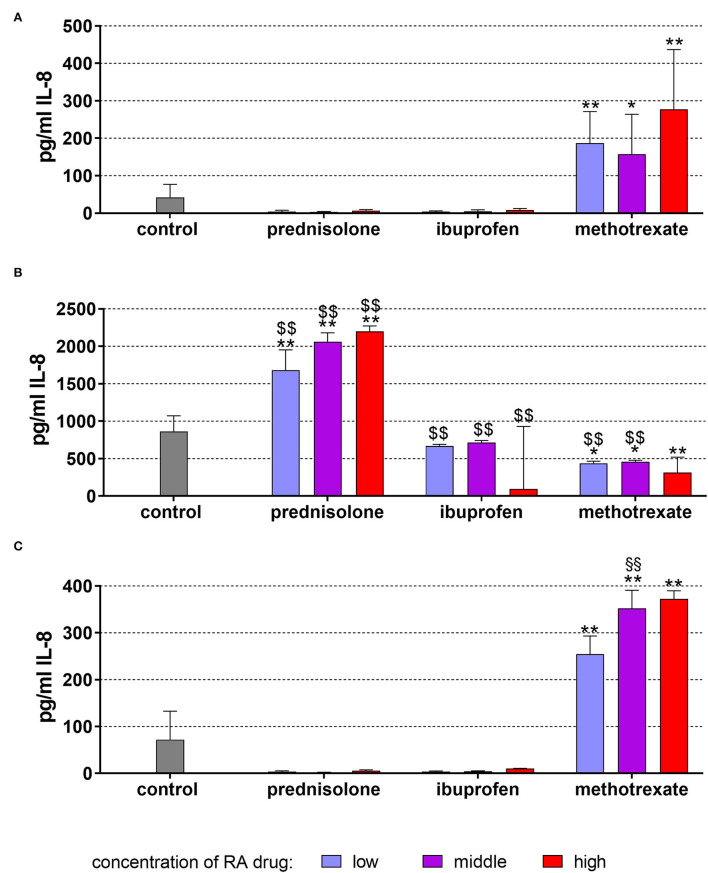
Released interleukin (IL)-8 by monocytic MONO-MAC-6 cells after 18 h of exposure of 20 ng/ml (low), 100 ng/ml (middle), 500 ng/ml (high) prednisolone, 2 μg/ml (low), 10 μg/ml (middle), 50 μg/ml (high) ibuprofen, 1 μg/ml (low), 5 μg/ml (middle), and 25 μg/ml (high) methotrexate **(A)** w/o microbial compounds, **(B)** with *Fusobacterium nucleatum* lysate, and **(C)** *Candida albicans* lysate. The columns represent the mean and the bars represent standard deviation. *p < 0.05 vs. control, **p < 0.01 vs. control. ^$$^*p* < 0.01 vs. respective group w/o microbial compounds.

Without stimulation, the cells were released in the mean of 42.47 ± 24.29 pg/ml IL-8 after 18 h of incubation. Prednisolone and ibuprofen in the three applied concentrations had no influence on IL-8 secretion. However, in presence of 1, 5, and 25 μg/ml methotrexate, the IL-8 levels were higher than in the controls (*p* = 0.001, *p* = 0.020, *p* < 0.001; Figure 1A).

The exposure to *F. nucleatum* lysate increased the level to 862.73 ± 209.00 pg/ml (*p* < 0.001). Higher levels of IL-8 vs. non-bacteria exposed controls were also detected in the presence of the drugs. Compared with *F. nucleatum* without drugs, ibuprofen did not change the level of IL-8, prednisolone increased the level further up to 2,201.46 ± 70.60 pg/ml (500 ng/ml *p* < 0.001). In contrast, methotrexate decreased the IL-8 release, but the levels were always still higher than without *F. nucleatum* (Figure 1B).

*C. albicans* lysate, however, did not significantly change the released amount of IL-8 (73.23±60.53 pg/ml). Methotrexate augmented the secretion (each *p* < 0.001), and at 5 μg/ml methothrexate, the IL-8 amount was higher with *C. albicans* lysate vs. cells without microbes (*p* = 0.006; Figure 1C).

**Figure 2:**
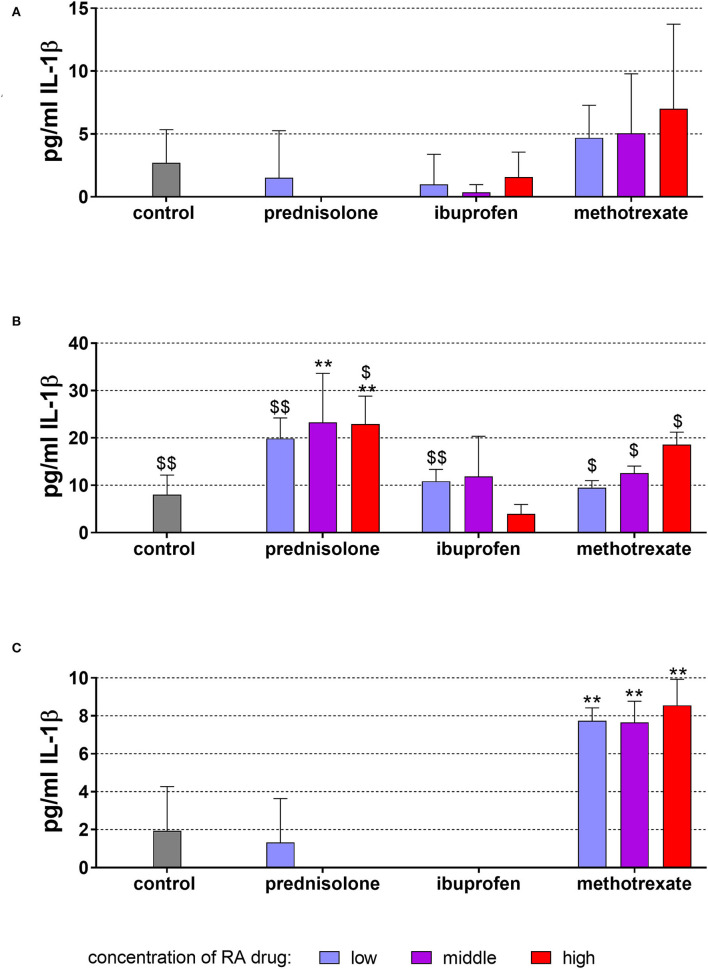
Released interleukin (IL)-1β by monocytic MONO-MAC-6 cells after 18 h of exposure of of 20 ng/ml (low), 100 ng/ml (middle), 500 ng/ml (high) prednisolone, 2 μg/ml (low), 10 μg/ml (middle), 50 μg/ml (high) ibuprofen, 1 μg/ml (low), 5 μg/ml (middle), and 25 μg/ml (high) methotrexate **(A)** w/o microbial compounds, **(B)** with *Fusobacterium nucleatum* lysate, and **(C)** *Candida albicans* lysate. The columns represent the mean and the bars represent standard deviation. *p < 0.05 vs. control, **p < 0.01 vs. control. ^$/$$^*p* < 0.05/*p* < 0.01 vs. respective group w/o microbial compounds.

The level of IL-1β released from MONO-MAC-6 cells without stimulation was in the mean of 2.70 ± 2.65 pg/ml after 18 h of incubation. This relatively low level was not changed further by any of the applied drugs (Figure 2A).

The exposure to *F. nucleatum* lysate increased the levels of IL-1β to 8.06 ± 4.10 pg/ml vs. unstimulated control (*p* < 0.001) (Figure 2B). Also in the presence of the RA drugs, the level of released IL-1β was most higher in the supernatants of the respective cells without microbes. Prednisolone in concentrations of 100 and 500 ng/ml elevated the amount of released IL-1β (*p* = 0.004, *p* = 0.006).

*C. albicans* lysate did not significantly change the released amount of IL-1β. Elevated levels of IL-1β were found in the presence of methotrexate in all three concentrations (each *p* < 0.001) (Figure 2C).

### PDL fibroblasts

**Figure 3:**
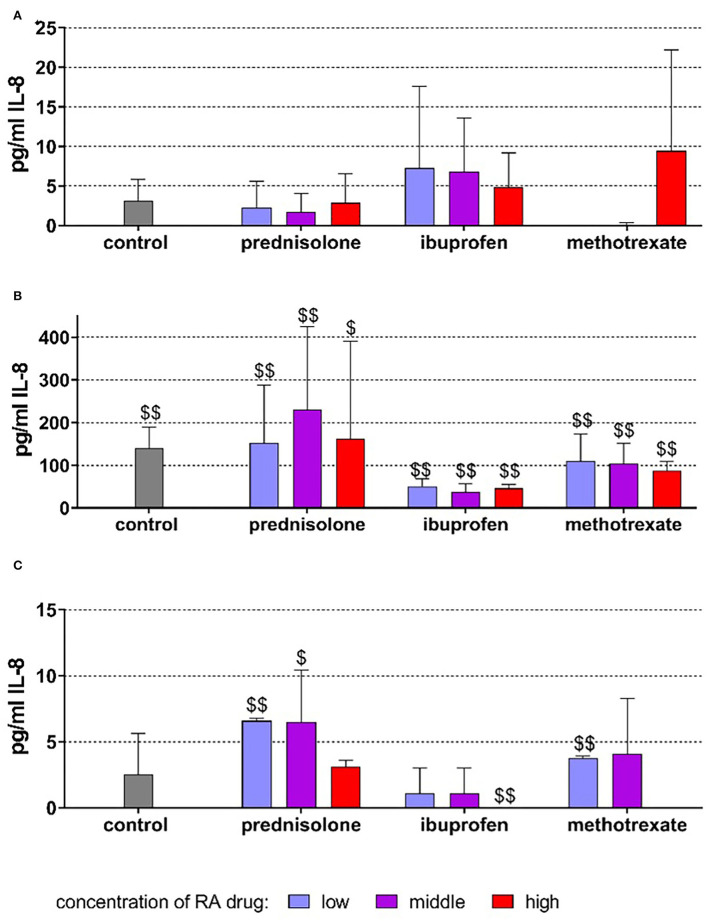
Released interleukin (IL)-8 by PDL fibroblasts after 18 h of exposure of 20 ng/ml (low), 100 ng/ml (middle), 500 ng/ml (high) prednisolone, 2 μg/ml (low), 10 μg/ml (middle), 50 μg/ml (high) ibuprofen, 1 μg/ml (low), 5 μg/ml (middle), and 25 μg/ml (high) methotrexate **(A)** w/o microbial compounds, **(B)** with *Fusobacterium nucleatum* lysate, and **(C)** *Candida albicans* lysate. The columns represent the mean and bars represent standard deviation. ^$/$$^*p* < 0.05/*p* < 0.01 vs. respective group w/o microbial compound.

Without stimulation, the cells released on average 3.17 ± 2.66 pg/ml IL-8 after 18 h of incubation. The addition of drugs did not statistically significantly modify the release of IL-8 (Figure 3A).

The exposure to *F. nucleatum* lysate increased the level to 140.33 ± 49.18 pg/ml (*p* < 0.001). The higher levels of IL-8 vs. non-bacteria exposed controls were also detected in the presence of the drugs (Figure 3B). *C. albicans* did not significantly change the released amount of IL-8 (2.52 ± 3.14 pg/ml). However, at low concentrations of prednisolone (*p* = 0.003) and methotrexate (*p* = 0.001), the amount of IL-8 was higher, and at 50 μg/ml, ibuprofen (*p* = 0.002) was lower than in cells without microbes (Figure 3C).

### TIGK cells

**Figure 4:**
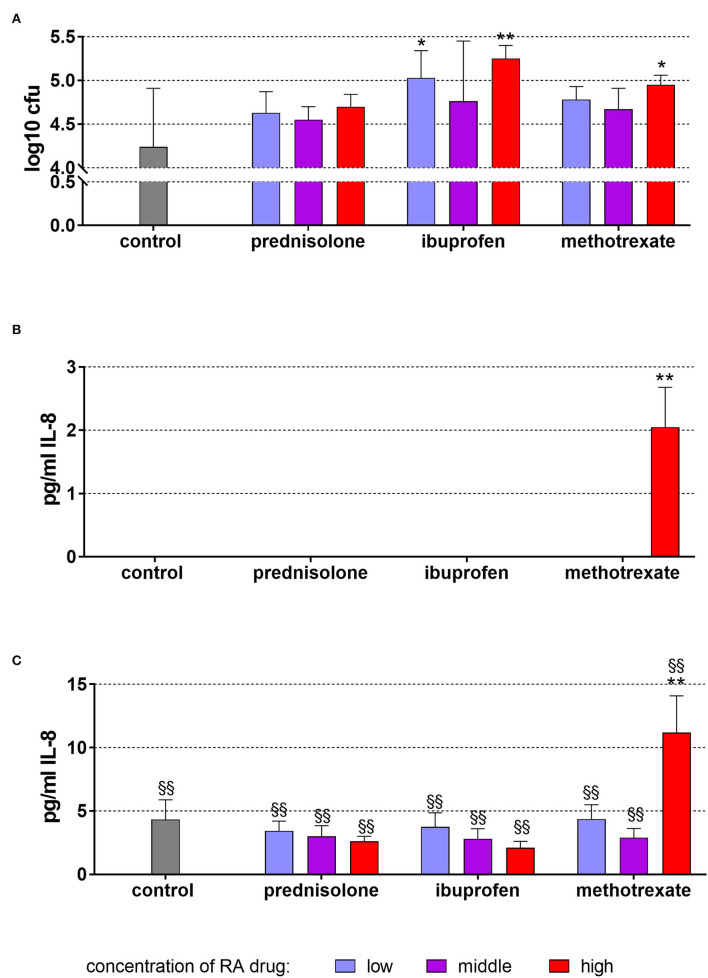
Attached (incl. invaded) *Candida albicans* to TIGK cells **(A)** and released IL-8 by TIGK cells after 6 h of exposure of 20 ng/ml (low), 100 ng/ml (middle), 500 ng/ml (high) prednisolone, 2 μg/ml (low), 10 μg/ml (middle), 50 μg/ml (high) ibuprofen, 1 μg/ml (low), 5 μg/ml (middle), and 25 μg/ml (high) methotrexate **(B)** w/o microbial compounds, and with **(C)** *Candida albicans*. The columns represent the mean and the bars represent standard deviation. *^/^**p < 0.05/*p* < 0.01 vs. control, ^*$*/*$$*^*p* < 0.05/*p* < 0.01 vs. respective group w/o microbial compound.

In these experiments, not microbial lysate but *C. albicans* cells were added. In the mean of 4.42 ± 0.67, log_10_ *C. albicans* cells attached to TIGK cells per well. In presence of RA drugs, always higher numbers were counted [0.31 log_10_ (100 ng/ml prednisolone) to 1.01 log_10_ (50 μg/ml ibuprofen)]. The differences were statistically significant for 2 μg/ml and 50 μg/ml ibuprofen (*p* = 0.013, *p* < 0.001) as well as for 25 μg/ml methotrexate (*p* = 0.048; Figure 4A).

Without microbial stimulus, there was only IL-8 detectable after the addition of 25 μg/ml methotrexate and 6 h of incubation (Figure 4B). The exposure to *C. albicans* increased the IL-8 levels without dependency on added RA drugs (Figure 4C). Also here, higher IL-8 levels were measured after 25 μg/ml methotrexate vs. *C. albicans* only (*p* < 0.001).

## Discussion

In this study, the influence of anti-rheumatic drugs with and without microbial stimulus on oral cells was analyzed. The addition of *F. nucleatum* lysate stimulated most strongly the release of proinflammatory cytokines by PDL fibroblast and monocytic cells. In contrast, the modification by the tested anti-rheumatic drugs was mostly minor.

Among the oral bacteria, *Porphyromonas gingivalis* is discussed as the most important link between rheumatoid arthritis and periodontitis [26]. *P. gingivalis* possesses a peptidyl-arginine deiminase that is able to citrullinate bacterial and host proteins [27]. Recently, it has been shown that the outer membrane vesicles of *P. gingivalis* may contain about 50 citrullinated proteins [28]. In our first experiments, *P. gingivalis* ATCC 33277 lysate (obtained by suspending bacterial cells, exposing them to ultrasonication, centrifugation, and filtrating the supernatant) was used. But in supernatants of PDL fibroblasts exposed to *P. gingivalis* without and with anti-rheumatic drugs, IL-8 was never measurable. This can be explained by the content of active gingipains which can cleave and degrade IL-8 [29]. Therefore, in the following experiments, *F. nucleatum* was used. Compared to periodontal health, the genus Fusobacterium is elevated in gingivitis [30] and it was most increased among all genera in periodontitis [31]. *F. nucleatum* is a strong inducer of inflammatory cytokines in gingival fibroblasts [32]. This was confirmed in the present study with regard to PDL fibroblasts and, in particular, monocytic cells.

When exposed to non-professional immune cells, that is, PDL-fibroblasts, *F. nucleatum* without the addition of anti-rheumatic drugs led to an increased release of IL-8. This increase, however, was smaller than in MONO-MAC-6-cells. *C. albicans* did not affect IL-8 expression. In the assays, we used *C. albicans* in the logarithmic growth phase (blastospores). In gingival fibroblasts, germinated *C. albicans* and not blastospores stimulated the release of IL-8 [33]. Among the three different included cell types, MONO-MAC-6 cells represent professional immune cells. These cells are potent cytokine producers except for interferon γ [34]. They release IL-1β and high levels of IL-8, in particular, after stimulation with *F. nucleatum* lysate. In contrast to *F. nucleatum, C. albicans* did not stimulate the release of IL-8. Our preparation method of *C. albicans* may suggest that the used lysate contained cell wall compartments. One cell wall component, the β-glucan has been shown to increase (together with LPS) the release of the anti-inflammatory cytokine IL-10 and do not affect the release of pro-inflammatory cytokines by human monocytes [35]. This may explain the missing influence of *C. albicans* on the release of pro-inflammatory mediators in our study. Supernatants from a *Candida*-biofilm did neither change IL-8 mRNA expression in blood cells nor the release of the cytokine into the supernatant [36]. Combined with mixed bacteria, however, there was an increase up to 86-fold of mRNA expression together with a high release [36]. It might be a limitation of the study that we did not stimulate the cells with a lysate obtained from a multi-species mixture.

MONO-MAC-6 cells released high levels of IL-1β after stimulation with *F. nucleatum* lysate, however, not after stimulation with *C. albicans*.

Glucocorticoids are widely used in the therapy of RA patients; they reduce inflammatory cell counts in tissues and decrease levels of inflammatory cytokines such as TNFα, IL-1 β, IL-6, and IL-17 [37]. Clinically, in RA patients, administration of prednisolone decreased synovial gene expression and protein level of IL-8 but not of IL-1β [38, 39]. In the present *in vitro* study, prednisolone, in general, did not influence the release of proinflammatory cytokines by any cells. Only together with *F. nucleatum*, prednisolone augmented the release of IL-8 and to a minor degree of IL-1β by MONO-MAC-6-cells.

Our results did not find an influence of ibuprofen on the release of IL-8 or IL-1β. This contrasts with a study on bovine fibroblast-like synoviocytes where ibuprofen lowered the secretion of IL-1β after lipopolysaccharide stimulation [40]. Clinically, in orthodontic patients, the use of ibuprofen did not influence the increase of IL-1β level in the gingival crevicular fluid after placement of elastomeric separators [41]. In the past, adjunctive ibuprofen was discussed in periodontal therapy. A 2-week administration lowered clinical signs of periodontal inflammation, but only as a short-term effect [42].

Methotrexate interacts with folate pathways, adenosine, leukotrienes, and cytokines, except for RA patients, it is used for chronic sarcoidosis patients [43]. In RA patients, the increased number of neutrophils can be decreased by methotrexate [44]. An increase in IL-1 expression and protein release by methotrexate has been reported in the monocytic U937 cell line which was correlated with activation of the JUN and FOS pathways [45]. Also in the present *in vitro* study, methothrexate increased the release without microbial stimulus; however, the release level was lowered after *F. nucleatum* stimulation. Thus, an ongoing inflammation induced or stimulated by bacteria associated with periodontal disease might be inhibited. In treatment-naïve RA patients, systemic inflammatory variables decreased after a 12-week intake of methotrexate alone or in combination with an anti-TNF-drug; however, there was no influence on periodontal inflammation which was quantified by the periodontal inflamed surface area (PISA) [46]. Clinically, inflammation and severity of periodontitis seem to be associated with the type of RA drugs. RA patients receiving methotrexate and rituximab (an antibody targeting B-cells) or leflunomide (an immunosuppressant) presented fewer sites with bleeding on probing than those receiving methotrexate and TNFα inhibitor [47]. In a recent evaluation of the antimicrobial activity of DMARDs against oral pathogens, methotrexate was the only DMARD to exert antimicrobial activity, and only one against *F. nucleatum* and *Viridans streptococci* [48]. With periodontitis, the level of IL-1β in GCF is controversially reported in patients with RA in comparison with those without, some studies reported higher levels in RA [16, 49], others lower levels in RA [50], and another study again did not find a difference [51]. All the RA patients were under respective RA treatment, details on the used drugs related to IL-1β levels were not reported. In a prospective study including RA patients, the levels of IL-1β and IL-8 decreased in the gingival crevicular fluid after TNFα-therapy [52].

The intake of anti-rheumatic drugs is designated to decrease RA disease activity. An analysis of salivary IL-β determined higher levels in periodontitis and RA patients than in periodontally and systemically healthy controls. The RA patients were under treatment with several DMRADs; subgrouping resulted in lower IL-1β levels in patients who received anti-TNFα therapy in comparison to the other RA patients [53].

In our recent analysis of microbiota in subgingival biofilm by using cultivation, surprisingly often yeasts were detected in RA patients which was not the case in periodontitis patients without RA [20]. Hence, we included *C. albicans* in that *in vitro* study. Methotrexate and ibuprofen increased the adhesion of *C. albicans* to gingival epithelial cells. Which mechanism, however, traffics the higher adhesion of *C. albicans* to host cells remains unclear. In *C. albicans*, Als3 functions as the adhesin to E-cadherin on oral epithelial cells and mediates endocytosis [54].

This study has several limitations. Only isolated cells were included. The *in vivo* immune response, however, is a result of concerted action of different cell types. Further, the model organism was *F. nucleatum*, but dental biofilm consists of hundreds of bacterial species. Then, the levels of cytokines were determined only after one single time point, whereas periodontal inflammation is a time-dependent process. Finally, lysates and not the whole bacteria were used.

Taken together, the release of inflammatory cytokines by professional and non-professional immune cells was mainly induced by bacterial stimuli. Among the tested anti-inflammatory drugs, methotrexate and prednisolone may interfere with the interaction of microorganisms with monocytic cells. Prednisolone increases the release of IL-8 after stimulation with *F. nucleatum*, whereas methotrexate decreased the level. Anti-inflammatory drugs increased the adherence of *C. albicans* to epithelial cells. Also, in patients with RA, the reduction of the microbial load in subgingival biofilm (biofilm removal) is of major importance to decrease the inflammatory response, however, the intake of inflammatory drugs may lead to untypical clinical signs of periodontitis in RA.

## Data availability statement

The raw data supporting the conclusions of this article will be made available by the authors, without undue reservation.

## Ethics statement

Ethical review and approval was not required for this study in accordance with the local legislation and institutional requirements. Written informed consent was obtained from all participants for their participation in this study.

## Author contributions

SE and ASt: conceptualization, methodology, and writing. ASt, CS, and GZ: experiments. SE and ASc: interpretation. SE: statistical analyses and supervision. All authors: review and editing. All authors have agreed to the published version of the manuscript.

## Funding

The study was entirely funded by the Department of Periodontology, University of Bern, Bern, Switzerland. Open access funding was provided by the University of Bern.

## Conflict of interest

The authors declare that the research was conducted in the absence of any commercial or financial relationships that could be construed as a potential conflict of interest.

## Publisher's note

All claims expressed in this article are solely those of the authors and do not necessarily represent those of their affiliated organizations, or those of the publisher, the editors and the reviewers. Any product that may be evaluated in this article, or claim that may be made by its manufacturer, is not guaranteed or endorsed by the publisher.

## References

[1] Kassebaum NJ Bernabe E Dahiya M Bhandari B Murray CJ Marcenes W. Global burden of severe periodontitis in 1990-2010: a systematic review and meta-regression. J Dent Res. (2014) 93:1045–53. 10.1177/0022034514552491 25261053 PMC4293771

[2] Van Dyke TE Bartold PM Reynolds EC. The nexus between periodontal inflammation and dysbiosis. Front Immunol. (2020) 11:511. 10.3389/fimmu.2020.00511 32296429 PMC7136396

[3] Llama-Palacios A Potupa O Sanchez MC Figuero E Herrera D Sanz M. Proteomic analysis of Fusobacterium nucleatum growth in biofilm versus planktonic state. Mol Oral Microbiol. (2020) 35:168–80. 10.1111/omi.12303 32558324

[4] Marchesan JT Girnary MS Moss K Monaghan ET Egnatz GJ Jiao Y. Role of inflammasomes in the pathogenesis of periodontal disease and therapeutics. Periodontol. (2020) 82:93–114. 10.1111/prd.12269 31850638 PMC6927484

[5] Palomino DC Marti LC. Chemokines and immunity. Einstein. (2015) 13:469–73. 10.1590/S1679-45082015RB3438 26466066 PMC4943798

[6] Finoti LS Nepomuceno R Pigossi SC Corbi SC Secolin R Scarel-Caminaga RM. Association between interleukin-8 levels and chronic periodontal disease: a PRISMA-compliant systematic review and meta-analysis. Medicine. (2017) 96:e6932. 10.1097/MD.0000000000006932 28562542 PMC5459707

[7] Grossi SG Zambon JJ Ho AW Koch G Dunford RG Machtei EE. Assessment of risk for periodontal disease. I Risk indicators for attachment loss. J Periodontol. (1994) 65:260–7. 10.1902/jop.1994.65.3.260 8164120

[8] Lee KH Choi YY. Rheumatoid arthritis and periodontitis in adults: using the Korean National Health Insurance Service-National Sample Cohort. J Periodontol. (2020) 91:1186–93. 10.1002/JPER.19-0311 31984496

[9] Sparks J. A. (2019). Rheumatoid arthritis. Ann. Intern. Med. 170:ITC1–16. 10.7326/AITC201901010 30596879

[10] An Q Yan W Zhao Y Yu K. Enhanced neutrophil autophagy and increased concentrations of IL-6, IL-8, IL-10 and MCP-1 in rheumatoid arthritis. Int Immunopharmacol. (2018) 65:119–28. 10.1016/j.intimp.2018.09.011 30312880

[11] Dai Y Hu S. Recent insights into the role of autophagy in the pathogenesis of rheumatoid arthritis. Rheumatology. (2016) 55:403–10. 10.1093/rheumatology/kev337 26342228

[12] Dayer JM. The saga of the discovery of IL-1 and TNF and their specific inhibitors in the pathogenesis and treatment of rheumatoid arthritis. Joint Bone Spine. (2002) 69:123–32. 10.1016/S1297-319X(02)00363-9 12027302

[13] Akram M Daniyal M Sultana S Owais A Akhtar N Zahid R. Traditional and modern management strategies for rheumatoid arthritis. Clin Chim Acta. (2021) 512:142–55. 10.1016/j.cca.2020.11.003 33186593

[14] Keysser G. Safety aspects of the treatment with glucocorticoids for rheumatoid arthritis. Z Rheumatol. (2021) 80:295–304. 10.1007/s00393-021-00972-x 33704557 PMC7948162

[15] Bender P Burgin WB Sculean A Eick S. Serum antibody levels against Porphyromonas gingivalis in patients with and without rheumatoid arthritis - a systematic review and meta-analysis. Clin Oral Investig. (2017) 21:33–42. 10.1007/s00784-016-1938-5 27561661

[16] Bender P Egger A Westermann M Taudte N Sculean A Potempa J. Expression of human and Porphyromonas gingivalis glutaminyl cyclases in periodontitis and rheumatoid arthritis-A pilot study. Arch Oral Biol. (2019) 97:223–30. 10.1016/j.archoralbio.2018.10.022 30399509 PMC6252109

[17] Abusleme L Hoare A Hong BY Diaz PI. Microbial signatures of health, gingivitis, and periodontitis. Periodontol. (2021) 86:57–78. 10.1111/prd.12362 33690899

[18] Ebbers M Lubcke PM Volzke J Kriebel K Hieke C Engelmann R. Interplay between *P. gingivalis*, F nucleatum and A actinomycetemcomitans in murine alveolar bone loss, arthritis onset and progression. Sci Rep. (2018) 8:15129. 10.1038/s41598-018-33129-z 30310087 PMC6181973

[19] Temoin S Chakaki A Askari A El-Halaby A Fitzgerald S Marcus RE. Identification of oral bacterial DNA in synovial fluid of patients with arthritis with native and failed prosthetic joints. J Clin Rheumatol. (2012) 18:117–21. 10.1097/RHU.0b013e3182500c95 22426587 PMC3888235

[20] Maldonado A Pirracchio L Imber JC Burgin W Moller B Sculean A. Citrullination in periodontium is associated with *Porphyromonas gingivalis*. Arch Oral Biol. (2020) 114:104695. 10.1016/j.archoralbio.2020.104695 32315811

[21] Laugisch O Wong A Sroka A Kantyka T Koziel J Neuhaus K. Citrullination in the periodontium–a possible link between periodontitis and rheumatoid arthritis. Clin Oral Investig. (2016) 20:675–83. 10.1007/s00784-015-1556-7 26264638 PMC5146953

[22] Eick S Goltz S Nietzsche S Jentsch H Pfister W. Efficacy of chlorhexidine digluconate-containing formulations and other mouthrinses against periodontopathogenic microorganisms. Quintessence Int. (2011) 42:687–700. 21842009

[23] Bischoff KB. Physiologically based pharmacokinetic modelling. In: Drinking Water and Health, Vol. 8. Pharmacokinetics in Risk Assessment (Washington, D.C.: National Academy of Science) (1987).

[24] Rainsford KD. Ibuprofen: pharmacology, efficacy and safety. Inflammopharmacology. (2009) 17:275–342. 10.1007/s10787-009-0016-x 19949916

[25] Magee MH Blum RA Lates CD Jusko WJ. Prednisolone pharmacokinetics and pharmacodynamics in relation to sex and race. J Clin Pharmacol. (2001) 41:1180–94. 10.1177/00912700122012733 11697751 PMC4207281

[26] Potempa J Mydel P Koziel J. The case for periodontitis in the pathogenesis of rheumatoid arthritis. Nat Rev Rheumatol. (2017) 13:606–20. 10.1038/nrrheum.2017.132 28835673

[27] Wegner N Wait R Sroka A Eick S Nguyen KA Lundberg K. Peptidylarginine deiminase from Porphyromonas gingivalis citrullinates human fibrinogen and α-enolase: implications for autoimmunity in rheumatoid arthritis. Arthritis Rheum. (2010) 62:2662–72. 10.1002/art.27552 20506214 PMC2941529

[28] Larsen DN Mikkelsen CE Kierkegaard M Bereta GP Nowakowska Z Kaczmarek JZ. Citrullinome of porphyromonas gingivalis outer membrane vesicles: confident identification of citrullinated peptides. Mol Cell Proteomics. (2020) 19:167–80. 10.1074/mcp.RA119.001700 31754044 PMC6944236

[29] Mikolajczyk-Pawlinska J Travis J Potempa J. Modulation of interleukin-8 activity by gingipains from Porphyromonas gingivalis: implications for pathogenicity of periodontal disease. FEBS Lett. (1998) 440:282–6. 10.1016/S0014-5793(98)01461-6 9872387

[30] Nowicki EM Shroff R Singleton JA Renaud DE Wallace D Drury J. Microbiota and metatranscriptome changes accompanying the onset of gingivitis. MBio. (2018) 9: e00518–75. 10.1128/mBio.00575-18 29666288 PMC5904416

[31] López-Martínez J Chueca N Padial-Molina M Fernandez-Caballero JA García F O'valle F. Bacteria associated with periodontal disease are also increased in health. Med Oral Patol Oral Cir Bucal. (2020) 25:e745–51. 10.4317/medoral.23766 32701927 PMC7648922

[32] Jang JY Song IS Baek KJ Choi Y Ji S. Immunologic characteristics of human gingival fibroblasts in response to oral bacteria. J Periodontal Res. (2017) 52:447–57. 10.1111/jre.12410 27558278

[33] Dongari-Bagtzoglou A Wen K Lamster IB. Candida albicans triggers interleukin-6 and interleukin-8 responses by oral fibroblasts in vitro. Oral Microbiol Immunol. (1999) 14:364–70. 10.1034/j.1399-302X.1999.140606.x 10895692

[34] Neustock P Brand JM Kruse A Kirchner H. Cytokine production of the human monocytic cell line Mono Mac 6 in comparison to mature monocytes in peripheral blood mononuclear cells. Immunobiology. (1993) 188:293–302. 10.1016/S0171-2985(11)80237-8 8225390

[35] Leonhardt J Gross S Marx C Siwczak F Stengel S Bruns T. Candida albicans β-glucan differentiates human monocytes into a specific subset of macrophages. Front Immunol. (2018) 9:2818. 10.3389/fimmu.2018.02818 30555483 PMC6284042

[36] Bhardwaj RG Ellepolla A Drobiova H Karched M. Biofilm growth and IL-8 & TNF-alpha-inducing properties of Candida albicans in the presence of oral gram-positive and gram-negative bacteria. BMC Microbiol. (2020) 20:156. 10.1186/s12866-020-01834-3 32527216 PMC7291589

[37] Ferreira JF Ahmed Mohamed AA Emery P. Glucocorticoids and rheumatoid arthritis. Rheum Dis Clin North Am. (2016) 42: 33–46, vii. 10.1016/j.rdc.2015.08.006 26611549

[38] Youssef PP Haynes DR Triantafillou S Parker A Gamble JR Roberts-Thomson PJ. Effects of pulse methylprednisolone on inflammatory mediators in peripheral blood, synovial fluid, and synovial membrane in rheumatoid arthritis. Arthritis Rheum. (1997) 40:1400–8. 10.1002/art.1780400807 9259419

[39] Gerlag DM Boyle DL Rosengren S Nash T Tak PP Firestein GS. Real-time quantitative PCR to detect changes in synovial gene expression in rheumatoid arthritis after corticosteroid treatment. Ann Rheum Dis. (2007) 66:545–7. 10.1136/ard.2006.059790 16984938 PMC1856037

[40] Maghsoudi H Hallajzadeh J Rezaeipour M. Evaluation of the effect of polyphenol of escin compared with ibuprofen and dexamethasone in synoviocyte model for osteoarthritis: an in vitro study. Clin Rheumatol. (2018) 37:2471–8. 10.1007/s10067-018-4097-z 29663159

[41] Kaya Y Alkan Ö Kömüroglu AU Keskin S. Effects of ibuprofen and low-level laser therapy on orthodontic pain by means of the analysis of interleukin 1-beta and substance P levels in the gingival crevicular fluid. J Orofac Orthop. (2021) 82:143–52. 10.1007/s00056-020-00254-2 33097977

[42] Taiyeb Ali TB Waite IM. The effect of systemic ibuprofen on gingival inflammation in humans. J Clin Periodontol. (1993) 20:723–8. 10.1111/j.1600-051X.1993.tb00697.x 8276982

[43] Maksimovic V Pavlovic-Popovic Z Vukmirovic S Cvejic J Mooranian A Al-Salami H. Molecular mechanism of action and pharmacokinetic properties of methotrexate. Mol Biol Rep. (2020) 47:4699–708. 10.1007/s11033-020-05481-9 32415503

[44] Perpétuo IP Caetano-Lopes J Rodrigues AM Campanilho-Marques R Ponte C Canhão H. Methotrexate and low-dose prednisolone downregulate osteoclast function by decreasing receptor activator of nuclear factor-κβ expression in monocytes from patients with early rheumatoid arthritis. RMD Open. (2017) 3:e000365. 10.1136/rmdopen-2016-000365 28955481 PMC5604603

[45] Olsen NJ Spurlock CF 3rd Aune TM. Methotrexate induces production of IL-1 and IL-6 in the monocytic cell line U937. Arthritis Res Ther. (2014) 16, R17. 10.1186/ar4444 24444433 PMC3978848

[46] De Smit MJ Westra J Posthumus MD Springer G Van Winkelhoff AJ Vissink A. Effect of anti-rheumatic treatment on the periodontal condition of rheumatoid arthritis patients. Int J Environ Res Public Health. (2021) 18:2529. 10.3390/ijerph18052529 33806304 PMC7967392

[47] Ziebolz D Rupprecht A Schmickler J Bothmann L Krämer J Patschan D. Association of different immunosuppressive medications with periodontal condition in patients with rheumatoid arthritis: Results from a cross-sectional study. J Periodontol. (2018) 89:1310–7. 10.1002/JPER.17-0616 29786138

[48] Kussmann M Obermueller M Spettel K Winkler S Aletaha D. *In vitro* evaluation of disease-modifying antirheumatic drugs against rheumatoid arthritis associated pathogens of the oral microflora. RMD Open. (2021) 7:e001737. 10.1136/rmdopen-2021-001737 34588273 PMC8483044

[49] Xiao F Li C Lin Y Peng Z Xu X Wen Y. Increased risk of periodontitis occurrence in patients with rheumatoid arthritis and its association with the levels of IL-1β and TNF-α in gingival crevicular fluid. Ann Palliat Med. (2021) 10:9078–87. 10.21037/apm-21-1782 34488393

[50] Miranda LA Islabão AG Fischer RG Figueredo CM Oppermann RV Gustafsson A. Decreased interleukin-1beta and elastase in the gingival crevicular fluid of individuals undergoing anti-inflammatory treatment for rheumatoid arthritis. J Periodontol. (2007) 78:1612–9. 10.1902/jop.2007.060520 17668981

[51] Cetinkaya B Guzeldemir E Ogus E Bulut S. Proinflammatory and anti-inflammatory cytokines in gingival crevicular fluid and serum of patients with rheumatoid arthritis and patients with chronic periodontitis. J Periodontol. (2013) 84:84–93. 10.1902/jop.2012.110467 22414257

[52] Üstün K Erciyas K Kisacik B Sezer U Pehlivan Y Öztuzcu S. Host modulation in rheumatoid arthritis patients with TNF blockers significantly decreases biochemical parameters in periodontitis. Inflammation. (2013) 36:1171–7. 10.1007/s10753-013-9652-9 23649513

[53] Mirrielees J Crofford LJ Lin Y Kryscio RJ Dawson DR 3rd Ebersole JL Miller CS. Rheumatoid arthritis and salivary biomarkers of periodontal disease. J Clin Periodontol. (2010) 37:1068–74. 10.1111/j.1600-051X.2010.01625.x 20880053 PMC2980566

[54] Phan QT Myers CL Fu Y Sheppard DC Yeaman MR Welch WH. Als3 is a Candida albicans invasin that binds to cadherins and induces endocytosis by host cells. PLoS Biol. (2007) 5:e64. 10.1371/journal.pbio.0050064 17311474 PMC1802757

