# Erratum: *In vitro* activity of anti-rheumatic drugs on release of pro-inflammatory cytokines from oral cells in interaction with microorganisms

**DOI:** 10.3389/froh.2023.1178207

**Published:** 2023-03-28

**Authors:** Frontiers Production Office

**Affiliations:** Frontiers Media SA, Lausanne, Switzerland

**Keywords:** periodontitis, rheumatoid arthritis, anti-inflammatory drugs, proinflammatory cytokines, oral cells

An Erratum on *In vitro* activity of anti-rheumatic drugs on release of pro-inflammatory cytokines from oral cells in interaction with microorganisms By Stähli A, Scherler C, Zappalà G, Sculean A and Eick S. (2022) Front. Oral. Health 3:960732. doi: 10.3389/froh.2022.960732

An omission to the funding section of the original article was made in error. The following sentence has been added: “Open access funding was provided by the University of Bern”.

The original version of this article has been updated.

